# Transcriptome Analysis of *Nautilus* and Pygmy Squid Developing Eye Provides Insights in Lens and Eye Evolution 

**DOI:** 10.1371/journal.pone.0078054

**Published:** 2013-10-16

**Authors:** Konstantinos Sousounis, Atsushi Ogura, Panagiotis A. Tsonis

**Affiliations:** 1 Department of Biology and Center for Tissue Regeneration and Engineering, University of Dayton, Dayton, Ohio, United States of America; 2 Ochadai Academic Production, Ochanomizu University, Tokyo, Japan; Texas A&M International University, United States of America

## Abstract

Coleoid cephalopods like squids have a camera-type eye similar to vertebrates. On the other hand, *Nautilus* (Nautiloids) has a pinhole eye that lacks lens and cornea. Since pygmy squid and *Nautilus* are closely related species they are excellent model organisms to study eye evolution. Having being able to collect *Nautilus* embryos, we employed next-generation RNA sequencing using *Nautilus* and pygmy squid developing eyes. Their transcriptomes were compared and analyzed. Enrichment analysis of Gene Ontology revealed that contigs related to nucleic acid binding were largely up-regulated in squid, while the ones related to metabolic processes and extracellular matrix-related genes were up-regulated in *Nautilus*. These differences are most likely correlated with the complexity of tissue organization in these species. Moreover, when the analysis focused on the eye-related contigs several interesting patterns emerged. First, contigs from both species related to eye tissue differentiation and morphogenesis as well as to cilia showed best hits with their Human counterparts, while contigs related to rabdomeric photoreceptors showed the best hit with their Drosophila counterparts. This bolsters the idea that eye morphogenesis genes have been generally conserved in evolution, and compliments other studies showing that genes involved in photoreceptor differentiation clearly follow the diversification of invertebrate (rabdomeric) and vertebrate (ciliated) photoreceptors. Interestingly some contigs showed as good a hit with Drosophila and Human homologues in *Nautilus* and squid samples. One of them, capt/CAP1, is known to be preferentially expressed in Drosophila developing eye and in vertebrate lens. Importantly our analysis also provided evidence of gene duplication and diversification of their function in both species. One of these genes is the Neurofibromatosis 1 (NF1/Nf1), which in mice has been implicated in lens formation, suggesting a hitherto unsuspected role in the evolution of the lens in molluscs.

## Introduction

The eye has been one of the wonders of evolution. That the common ancestor of all eyes must have been a cell (or few cells) that responded to light is more or less an accepted notion. During evolution though many different types of eyes have appeared including mirror, pinhole, camera-type and compound eyes. It is, however, very intriguing that the camera-type eye, a feature of all vertebrates, can be also found in invertebrates. Have all eyes evolved from a prototype eye or have they been evolved independently many times? The ideas on the monophyletic origin of eyes have been bolstered by regulation of their morphogenesis by pax-6 (and its homologues in invertebrates), thus heralded as the eye master gene in all species [1]. However, an important difference underlies the evolution of photoreceptors. Invertebrates have rhabdomeric photoreceptors with microvillar and are located towards the direct light. On the other hand, vertebrates have ciliary photoreceptors with disks stacked together and are embedded in the retina away from the direct light [2]. This is an important difference, which might infer independent evolution of photoreceptors, even though such more complex scenario is also compatible with pax-6 expression. 

An interesting case in eye evolution can be found in molluscs. All cephalopods, except Nautiloids, have a lens-baring camera-type eye. *Nautilus* has a pinhole eye, lacking lens and cornea. Thus the retina of *Nautilus* is basically open to the environment that is seawater. The small opening is required in order for the vision not to be blurry, however, this reduces the visual information [3–6]. The loss of the lens in *Nautilus* is probably due to the fact that *Nautilus* evolved to be a scavenger and opportunistic predator with a shell, actually keeping the ancestral characteristics of the cephalopod lineage. *Nautilus* can withdraw inside the shell for protection from predators and a hard lens might not have been the best tissue for this task. On the other hand, a camera-type eye provides significant perception of the surroundings which can be utilized for predation. This is hypothesized to be the driving force for positive selection of camera-type eyes. 

Thus, examination of gene expression in the developing eye of *Nautilus* and its relative, the pygmy squid must provide important and crucial information pertaining to the mechanisms that led to loss of lens in *Nautilus*. Recently the transcriptome of both species has been established and assembled de novo [5]. In the present paper this information is analyzed thoroughly to compare gene expression in hope that it will provide insights into the evolution of eye elements in invertebrates and vertebrates. Indeed, we have found interesting molecular signatures pertaining photoreceptor evolution as well as selection of genes via duplication.

## Methods

### Data collection information

Raw sequencing reads were obtained from the DNA Data Bank of Japan (DDBJ; ID:DRA000453)[5]. These data correspond to developing eyes from *Idiosepius paradoxus* (pygmy squid) embryos at stage 24 and *Nautilus pompilius* embryos 30 days in development [5].

### Functional annotation and enrichment of Gene Ontology terms

De novo assembly was performed using Phred/Phrap. Read alignments to the reference were performed with Novocraft novoalign v2.06.09 package and expression of contigs was represented as normalized, based on samples’ reads, average coverage. De novo assembly and expression calculation was performed by Cofactor Genomics Inc. and as reported by us [5]. Only contigs that were more than 100bp were used for the following steps. *Nautilus* and pygmy squid (mentioned as squid in the rest of the text) contigs were annotated using BLAST2GO program against the non-redundant (nr) database with e-value cutoff of 1E-10 [7]. Gene Ontology (GO) terms were found using the annotation with augmentation tool of BLAST2GO with default parameters [8]. Custom perl scripts were used to extract differential regulated contigs. Contigs that had an average coverage of 10 or more and were expressed more than two times between the two samples were used. Fisher’s exact test corrected for multiple selections (feature available in BLAST2GO) were used to identify over-represented GO terms between the extracted *Nautilus* and squid contigs (FDR < 0.05).

### Extracting eye-related contigs

Human and fly eye-related genes were found from UniProt (http://www.uniprot.org/)[9] by searching for the following GO terms “eye”, “lens”, “retina”, “photoreceptor”, “R1/R6”, “R2/R5”, “R3/R4”, “R7”, “R8” and organism “*Homo sapiens*” or “*Drosophila melanogaster*”. All the protein results from these searches were downloaded as a fasta file. Human and Drosophila fasta files were compared using the BLAST tool[10] to the *Nautilus* and squid transcriptomes making the raw eye-related contigs for each of the organism. Next the complete Human and Drosophila proteomes were downloaded from UniProt and were combined to a single file. This file was compared using the BLAST tool to the *Nautilus* and squid transcriptomes finding the best hits of each of the contigs. These results were compared to the results of the raw eye-related contigs to ensure that they are the best eye-related hits. Comparisons between the groups were performed using custom perl scripts.

### Duplication and selection events

**Figure 1:**
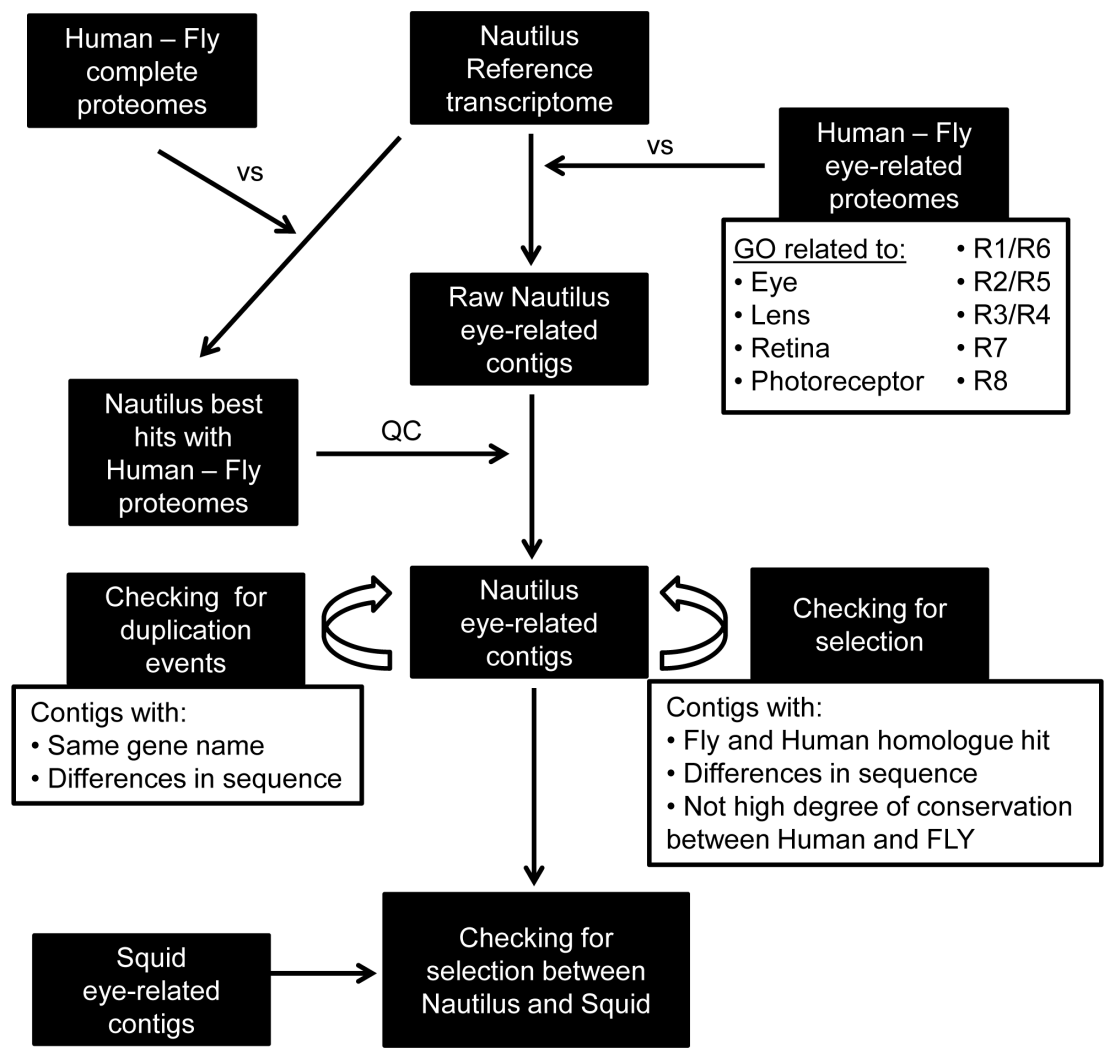
General scheme of work flow. *Nautilus* transcriptome was compared to Human and Drosophila eye-related proteomes taken from UniProt based on GO terms. The raw eye-related contigs were compared with the results of the comparison between the transcriptome and the complete Human and Drosophila proteomes in order to ensure the best hits of these contigs (quality control: QC). The eye-related contigs were analyzed for duplication events and gene selection. Same procedure was used for the squid transcriptome. The final eye-related contigs were also compared between the *Nautilus* and squid.

The analysis of gene duplication was based on the fact that if there are two or more contigs in *Nautilus* or squid transcriptome and share the same best hit then it is probable that there is a duplication of this gene. Evaluation of the candidates also included the length of the contigs (>100bp) and the similarity between the two contigs (<95%). For the present study all the candidates were tested manually using the BLAST tool. The analysis of differential selection of genes was based on the fact that if there are two contigs, one expressed in the *Nautilus* transcriptome and one expressed in the Squid transcriptome, and they share the same hit in the Drosophila and Human homologues then this gene is a candidate for differential selection after the diversification of the *Nautilus* and the squid. All the candidates were tested manually using the BLAST tool. Candidates needed to share a hit in a region that is not conserved between Human and Drosophila. A general scheme of these comparisons is presented in Figure 1. Alignments and Neighbour-joining distances were performed using ClustalW2 with default parameters [11]. Contigs were trimmed to match the hit on the fly or human homologue gene and they were checked again for their best hits in Drosophila and Human homologues using the BLAST tool. BLASTN tool was used for comparisons between contigs, BLASTX and TBLASTN tools were used for comparisons between *Nautilus* and squid transcriptomes, and Human and Drosophila proteomes. Drosophila and Human homologues were found with HomoloGene (http://www.ncbi.nlm.nih.gov/homologene) and GeneCards [12]. Sequences for individual proteins were obtained from UniProt. Phylogenetic tree was created using ClustalW2 with protein sequences from *Nautilus* NF1 contig (comp83316_c0_seq1), *Nautilus* Nf1 contig (comp63708_c0_seq1), squid NF1 contig (comp475299_c0_seq1), squid Nf1 contig (comp304995_c0_seq1), Human NF1 (NP_001035957.1), Human NF2 (CAG30416.1), Drosophila Nf1 (AAB58976.1) and Drosophila Merlin (AAB08449.1).

## Results and Discussion

### Next generation RNA sequencing in Nautilus and Squid developing eyes

79,254,130 and 91,319,864 reads were obtained online from *Nautilus* and squid developing eye samples, respectively. From those reads, 36,392,640 (45.9%) and 10,926,326 (12%) were aligned to the de-novo assembled reference transcriptome. Sequences that were more than 100bp were further used. Expression of contigs was calculated as normalized average coverage, and a cutoff of 10 was used in order to obtain genes that were adequately expressed and to rule out false positives. Contigs expressed at least two-fold more between the *Nautilus* and squid transcriptome were considered to be differentially expressed. These sequences were annotated using the BLAST2GO program. From the total 64,335 and 38,148, 7,219 (11.2%) and 3,941 (10.3%) contigs were annotated and associated with Gene Ontology terms in the *Nautilus* and squid samples, respectively. These data are comparable to the assembly created before [5]. In addition, we note here that the squid sample yielded around 10 million more reads than the *Nautilus* sample but these reads failed to contribute to the de-novo assembly and, subsequently, to the alignment. This resulted to the better coverage of the *Nautilus* sample indicated by the better representation in the nr database, however, most of the genes associated with eye development have been found in both the samples (Table 1; as also seen before [5]). The percent of annotated genes was comparable between the two samples. The high cutoff of the normalized average coverage that was set ensues that the genes used for Gene Ontology enrichment are actually expressed in the different samples and are not due to the better coverage of the one sample versus the other. 

**Table 1:** Selected *Nautilus* or squid eye-related contigs having best hits with their Human or Drosophila homologues divided based on their function.

Group	Human				Drosophila		
Eye patterning	SOX2	PAX6	MITF	OTX2	dac	-	-
	SIX3	NR2E1	PAX2	MAB21L2	-	-	-
	VAX1	TFAP2A	MEIS1	PBX1	-	-	-
Transport	VPS18	AP1M1	AP3D1	AP2B1	AP-1sigma	Arf79F	-
	DNAJC6	VPS16	ARFGAP2	VPS28	-	-	-
	AP1G1	AP1B1	-	-	-	-	-
CK-related in PR	CEP290	IQCB1	BBS1	BBS2	mbt	shh	dsh
	BBS4	MKKS	NPHP3	TTC8	Abi	arm	yrt
	NPHP4	NPHP1	RPGRIP1	AHI1	Sra-1	Patj	msn
	MAK	IFT57	DKFZp564L232	KIF3A	Src42A	stan	Moe
	IFT140	IFT52	IFT88	CETN3	mts	DKFZp686E0752	Cam
	PFDN5	CETN2	WHRN	OCRL	-	-	-
	CEP95	PROM1	MYO7A	USH1G	-	-	-
	USH1C	GPR98	-	-	-	-	-
EGFR pathway	-	-	-	-	cnk	csw	S
	-	-	-	-	Cbl	rg	sty
Notch pathway	-	-	-	-	rn	N	nct
	-	-	-	-	fng	p120ctn	Egm
	-	-	-	-	AP-47	sca	frc
	-	-	-	-	neur	rg	svp

Abbreviations: CK: Cytoskeleton, PR: Photoreceptors

### GO differences between Nautilus and Squid developing eyes

**Figure 2:**
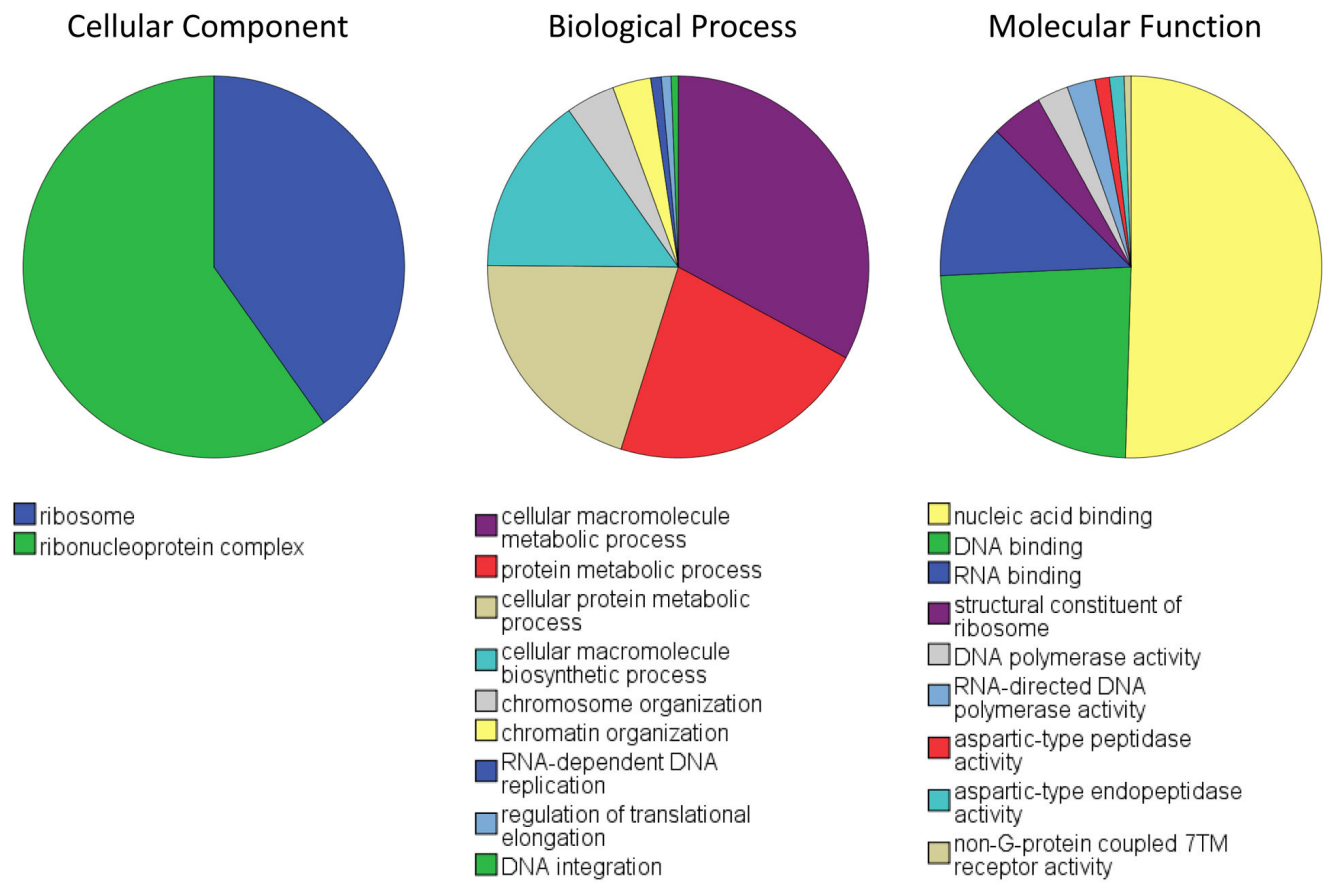
GO terms that are over-represented in the developing eye of the squid versus the developing eye of *Nautilus*. Results generated using fisher’s exact test corrected with multiple selection between contigs expressed at least two-fold in the squid sample versus the *Nautilus* sample.

**Figure 3:**
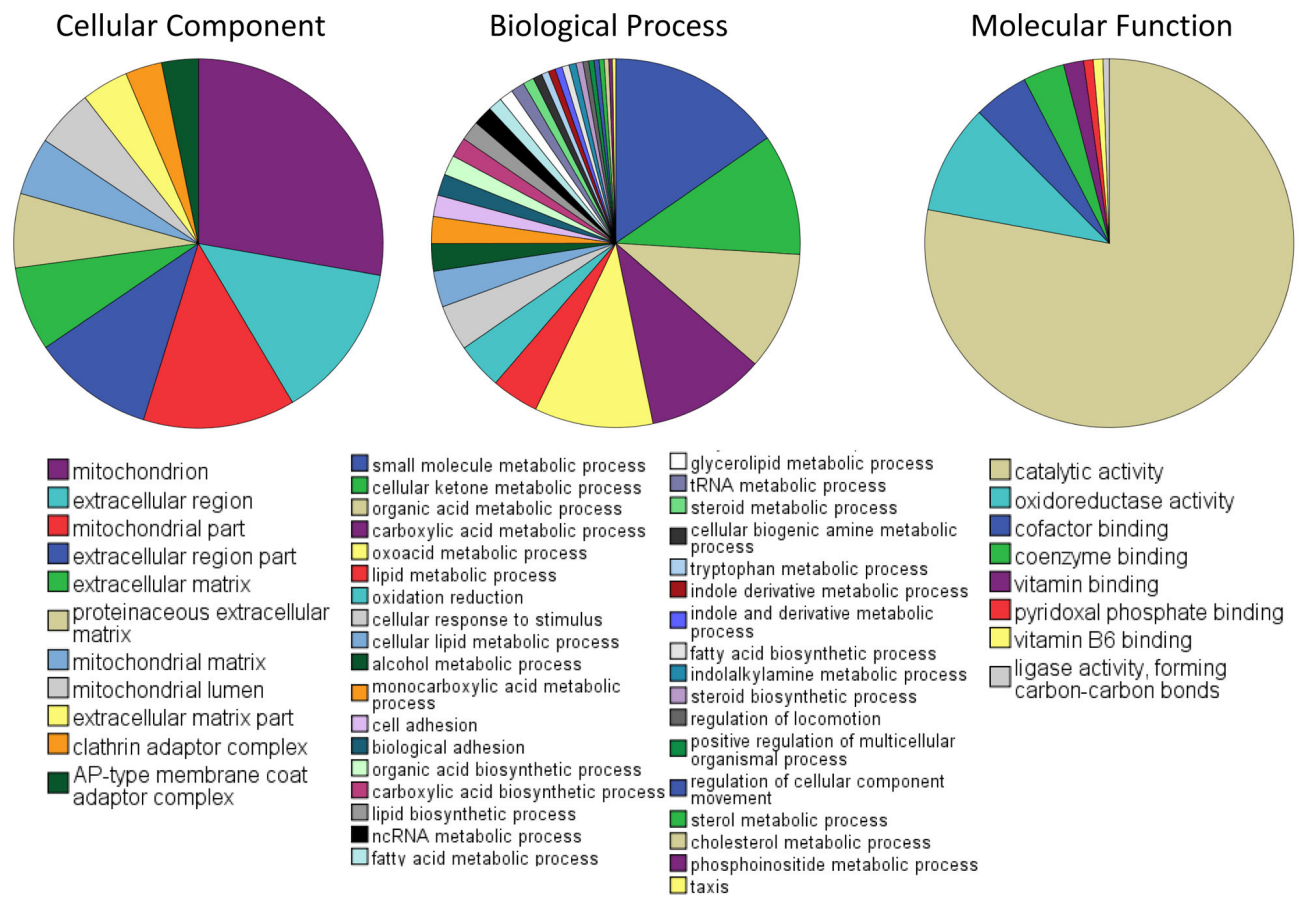
GO terms that are over-represented in the developing eye of the *Nautilus* versus the developing eye of the squid. Results generated using fisher’s exact test corrected with multiple selection between contigs expressed at least two-fold in the *Nautilus* sample versus the squid sample.

First we asked what kind of patterns was different between the two species. In order to do this we considered genes that are at least 2-fold higher in one species versus the other. Fisher’s exact test corrected for multiple selections was used in the GO terms that fulfilled the above criterion (Table S1). The GO terms that were found to contain genes over-expressed in the squid developing eyes are presented as pies (Figure 2). What is remarkable with this analysis is that the over-expressed genes dominate the nucleic acid-binding terms. In contrast the patterns in *Nautilus* were different with GO terms dominated by genes implicated in metabolic and catalytic function as well as by genes involved in cell adhesion (Figure 3). We believe that these differences are due to the fact that the camera-type eye of the developing squid is undergoing a more complex and faster morphogenesis than *Nautilus* [13].

### Differences between eye-related genes expressed in the developing eyes of Nautilus and squid

Since there are differences in gene expression between *Nautilus* and squid developing eyes, we were compelled to analyze the differences from a gene evolution perspective. We were interested to know whether a particular contig (from *Nautilus* or squid) had a better hit with its Human homolog or with its Drosophila homologue. All the sequences (from both species) were compared to proteins that have a GO term related to eye, photoreceptor, retina, lens, R1/R6, R2/R5, R3/R4, R7 or R8 from human (*Homo sapiens*) or Drosophila (*Drosophila melanogaster*) origin. We used these two organisms because they have the best annotated genomes of a vertebrate and invertebrate, respectively. *Nautilus* and squid contigs were compared to human and fly proteins as seen in the work flow in Figure 1. The results are summarized in Table S2. 

The analysis revealed an interesting pattern. We found that genes involved in eye morphogenesis have the best hit with their Human homologues. These are: PAX6, SOX2, MITF, OTX2, SIX3 NR2E1, PAX2, MAB21L2, VAX1, TFAP2A, MEIS1, and PBX1 (Table 1). Also, set of proteins that act as complexes such as (BBS1, BBS2, BBS4, MKKS, NPHP3, TTC8), (NPHP1, NPHP4, RPGRIP1, AHI1, MAK) and (IFT57, DKFZp564L232, IFT140, IFT52, IFT88) all playing role in cilium formation have a best hit with their human homologues. Interestingly, genes that are involved in transport (axon growth and regulation of pigment molecules during eye development) have best hit with their human homoloques. As it has been previously reported SIX3 and downstream factors are not expressed at all in *Nautilus* and this might indicate their role in the evolution of the pinhole eye in Nautilus [5].

On the other hand, cytoskeletal genes involved in photoreceptors have a very interesting combination of human and fly best hits. Genes involved in cell polarity and planar cell polarity, which mainly involve rearrangement and regulation of cytoskeleton, have a best hit with the Drosophila homologues while genes involved in centrioles and other cytoskeletal components, like the Bardet-Biedl syndrome complex, have a best hit with the Human homologues. These results clearly reflect the morphology of rhabdomeric versus ciliary photoreceptors (Table 1). Interestingly, genes involved in the same signaling pathway such as in Notch and EGFR pathways have best hits with their Drosophila homologues and the function of these genes is required for the correct specification and patterning of the different cell types during Drosophila eye development [14]. 

This analysis clearly indicates that eye morphogenesis genes have not really changed much over the course of evolution and that have been always used in different eye types. On the other hand, photoreceptor-related genes have either been selected for rabdomeric or ciliary photoreceptors, indicating a possible independent evolution. In addition, eye morphogenesis in arthropods seems to have diversified from a conserved group of proteins that could account for a vertebrate camera-type eye. This diversification could have led to changes also in the circuits of gene interactions as found from experiments with fruit flies including the duplication of the ortholog of the human PAX6, *eyeless* [15,16]. 

### Are there any genes selected for lens evolution in squid?

**Figure 4:**
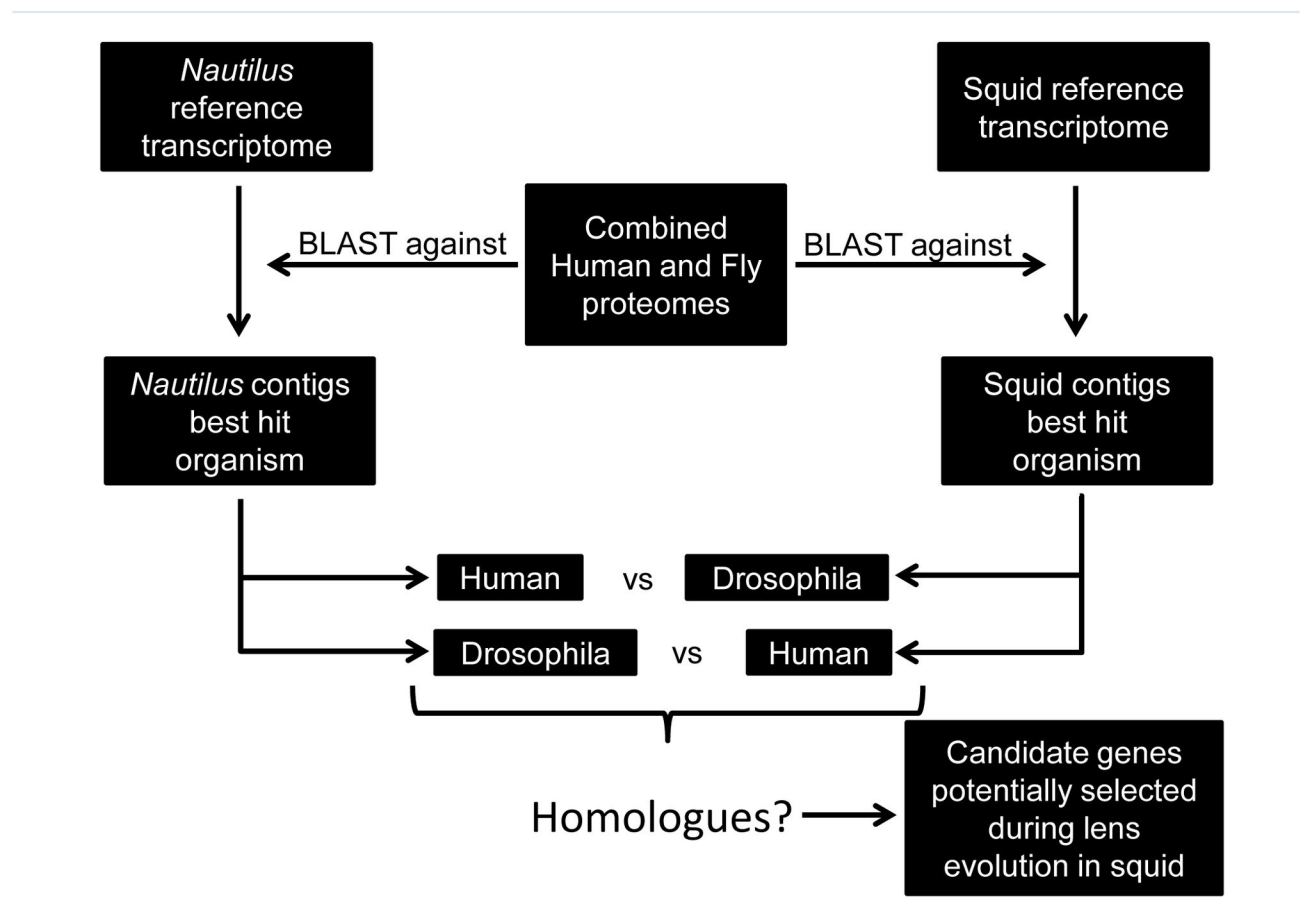
Work flow to find candidates selected for lens evolution in squids. *Nautilus* and squid transcriptomes were compared with a combined Human and Drosophila proteome. Contigs between *Nautilus* and squid that shared a homologue gene in Drosophila and Human were further analyzed.

**Figure 5:**
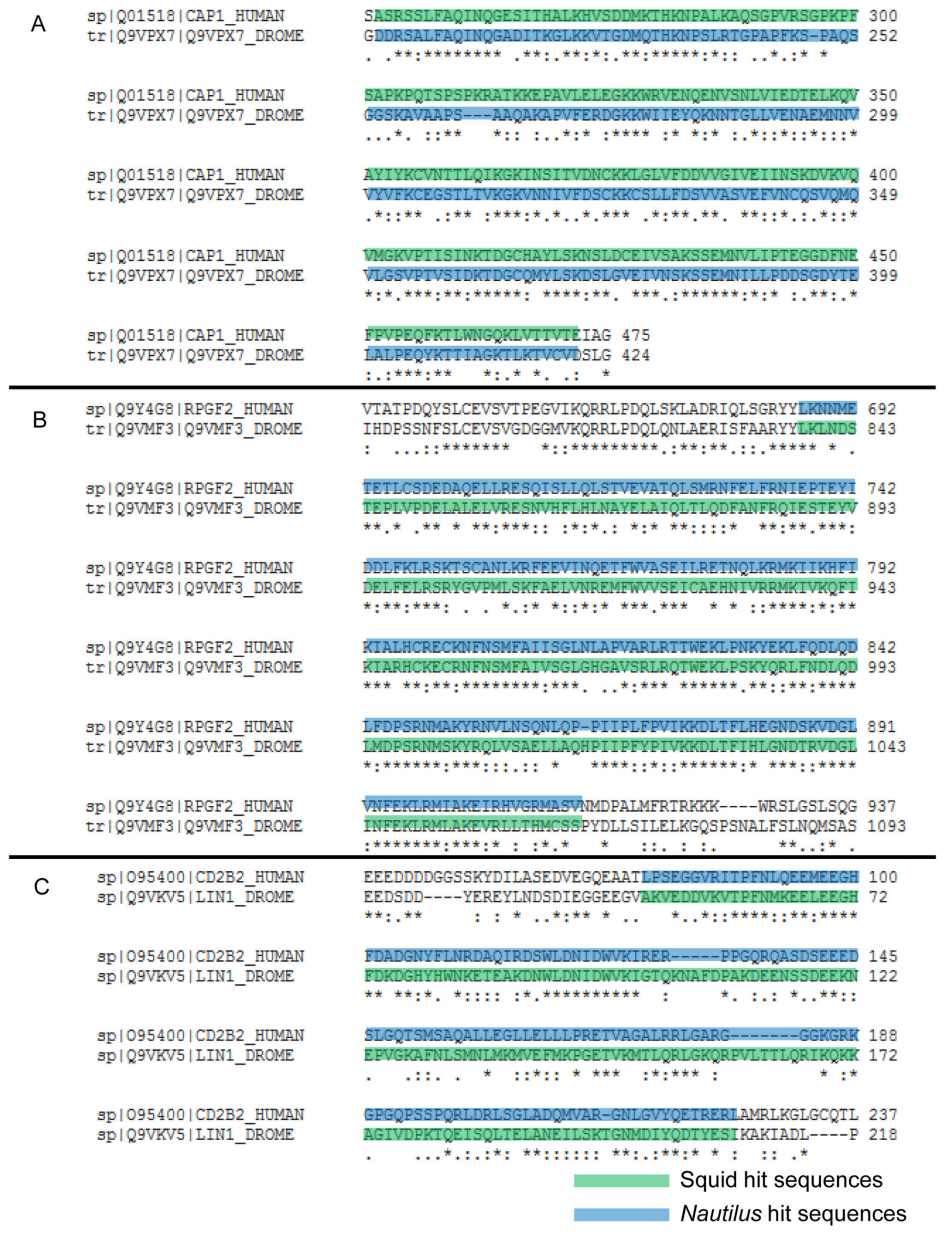
Alignments of the three gene pairs found to be selected for lens evolution in squids. Drosophila and Human homologue protein sequence were aligned using ClustalW2 to show conserved regions. Highlighted areas indicate the squid or *Nautilus* hit sequences with their counterpart homologues in Human or Drosophila. Proteins are labeled with their UniProt entry. A. CAP1/capt gene pair alignment. Squid has a best hit with the Human homologue (CAP1; CAP1_HUMAN) while *Nautilus* has a best hit with the Drosophila homologue (capt; Q9VPX7_DROME) in the same region. B. RAPGEF2/Gef26 gene pair alignment. Squid has a best hit with the Drosophila homologue (Gef26; Q9VMF3_DROME) while *Nautilus* has a best hit with the Human homologue (RAPGEF2; RPGF2_HUMAN) in the same region. C. CD2BP2/CG5198 gene pair alignment. Squid has a best hit with the Drosophila homologue (CG5198; LIN1_DROME) while *Nautilus* has a best hit with the Human homologue (CD2BP2; CD2B2_HUMAN) in the same region. Asterisk(*) indicate sequence identity, colon(:) indicates strongly similar properties and period(.) indicates weakly similar properties (ClustalW2).

**Figure 6:**
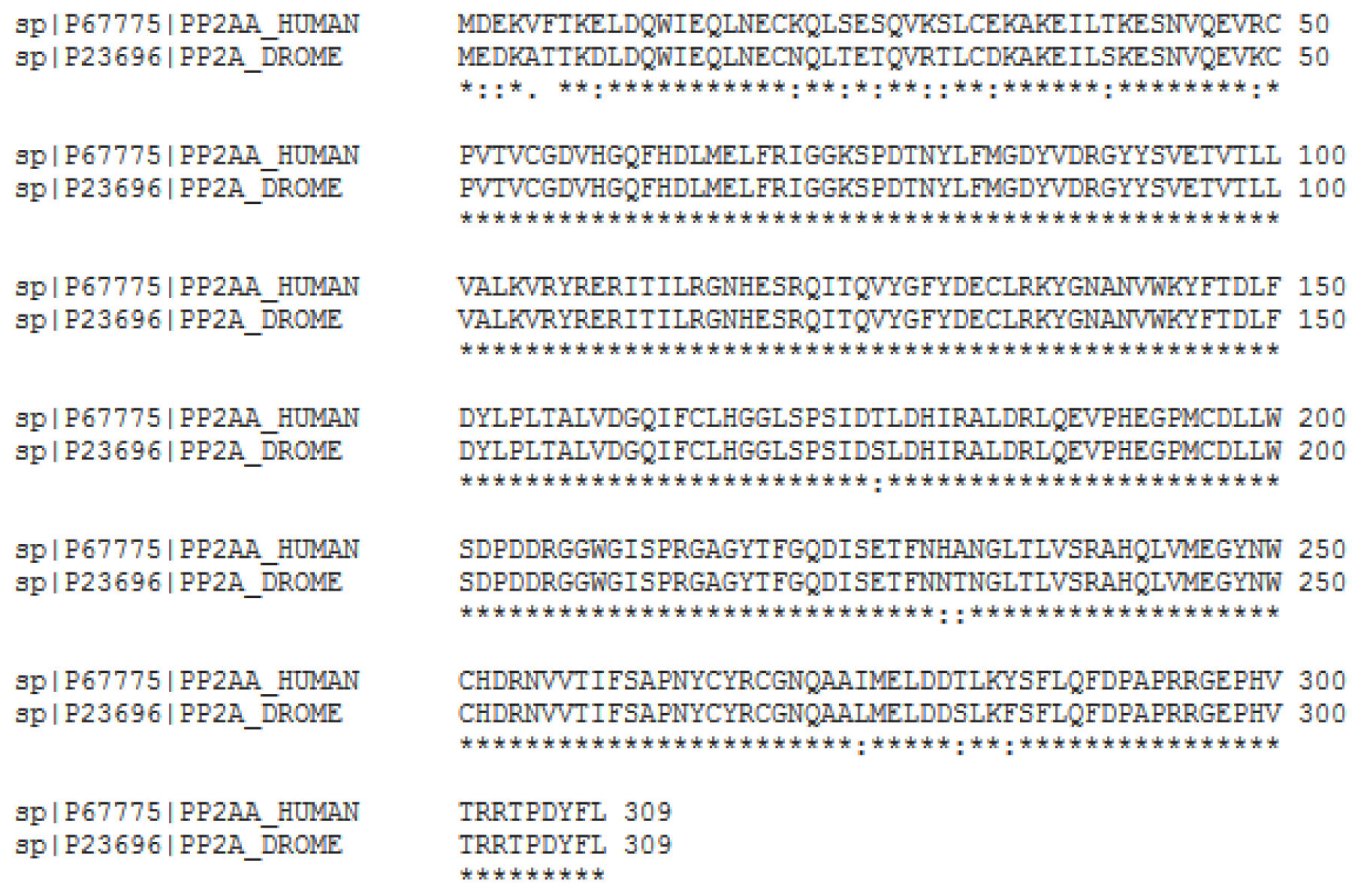
An example of Human/Drosophila conserved protein. PPP2CA/mts pair was found in *Nautilus* and squid as a potential candidate for lens evolution. These pairs were discarded from our analysis due to the high conservation of the Human and Drosophila proteins. Asterisk(*) indicate sequence identity, colon(:) indicates strongly similar properties and period(.) indicates weakly similar properties (ClustalW2).

Based on the previous analysis, certain genes are more related to vertebrates (eye morphogenesis genes, ciliary photoreceptor genes), while other are more related to invertebrates (rabdomeric photoreceptors, members of Notch and EGFR pathways). But are there any contig pairs (from *Nautilus* and squid) that have best hit with either Human or Drosophila homologues? To answer this question, we compared the *Nautilus* transcriptome with the combined Human and Drosophila proteome and we did the same with the squid transcriptome (Figure 4). If the one analysis (Nautilus versus Human and Drosophila) produced a best hit of “X” gene with Drosophila, and the other (Squid versus Human and Drosophila) produced a best hit of the same gene with the Human homologue, then we compared these two contigs from *Nautilus* and squid. These might indicate that certain genes were selected for lens evolution in squid. Potential candidates were investigated based on the length of the contig, the alignment to the Drosophila or Human homologue and the conservation between the Drosophila and Human homologue genes. We initially found 26 potential gene pairs from *Nautilus* and squid transcriptomes that have hits with the same Human and Drosophila gene homologue (Table S3). Only three gene pairs passed our evaluation criteria, CAP1/capt, RAPGEF2/Gef26 and CD2BP2/CG5198 (Figure 5). The other gene pairs were highly conserved between Human and Drosophila or aligned in conserved regions of the homologue genes (Figure 6). Figure S1, Figure S2 and Figure S3 show the alignments of the three gene pairs that passed our evaluation criteria. The Neighbour-joining distances are 0.35023, 0.29123 and 0.35455 for CAP1/capt, RAPGEF2/Gef26 and CD2BP2/CG5198 respectively. Interestingly, studies have shown that CAP1 is found to be expressed preferentially in the lens [17], while capt have been found to play role in eye morphogenesis in Drosophila [18]. Since CAP1 is found in squid and capt in *Nautilus*, we reasoned that this selection serves the function of CAP1 in the squid lens. 

### Evidence for gene duplication and selection for eye evolution

**Figure 7:**
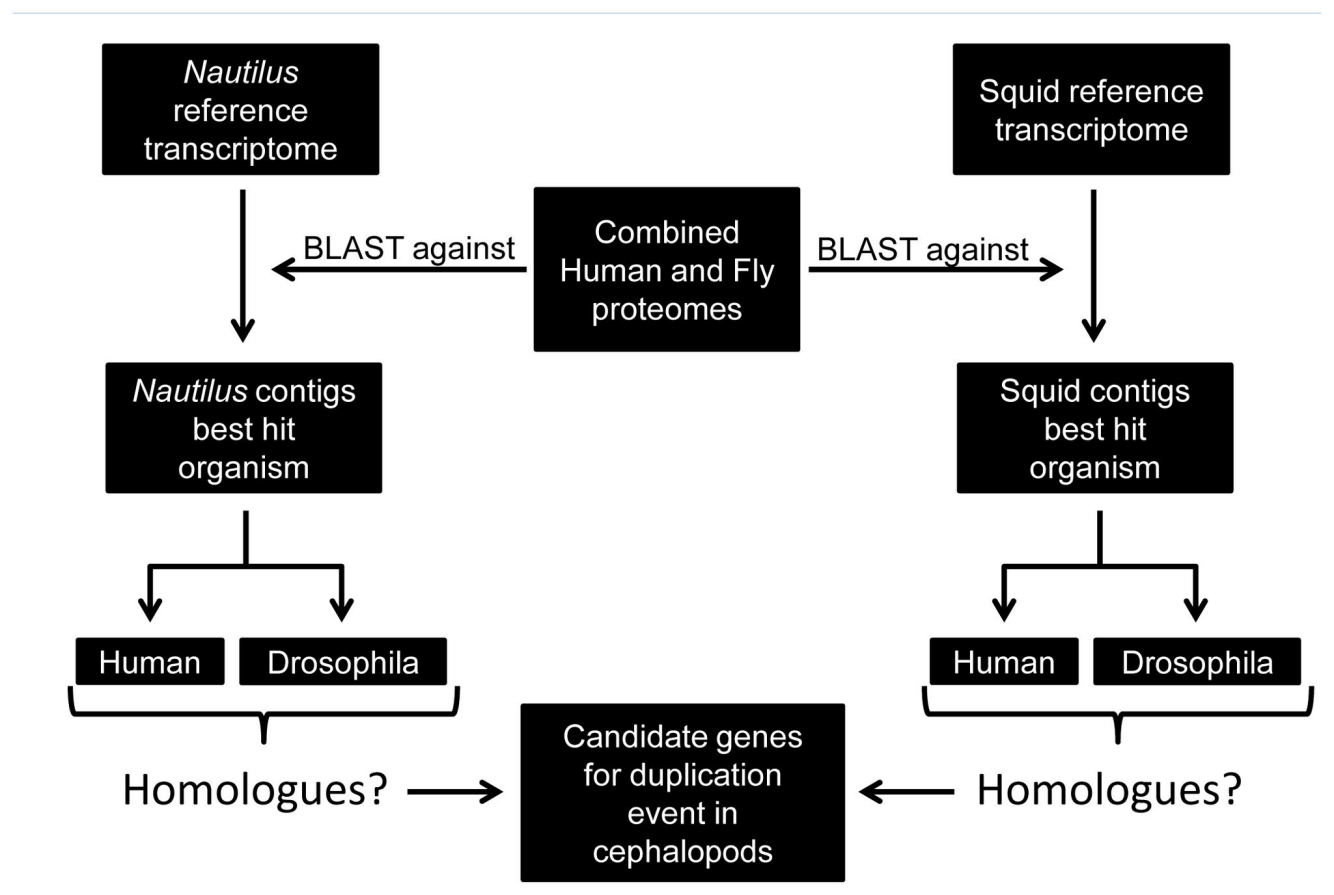
Work flow to find candidate genes that were duplicated and selected for eye evolution in cephalopods. *Nautilus* and squid transcriptome were compared with a combined Human and Drosophila proteome. Contigs in the Nautilus or squid that shared a homologue gene in Drosophila and Human were further analyzed.

**Figure 8:**
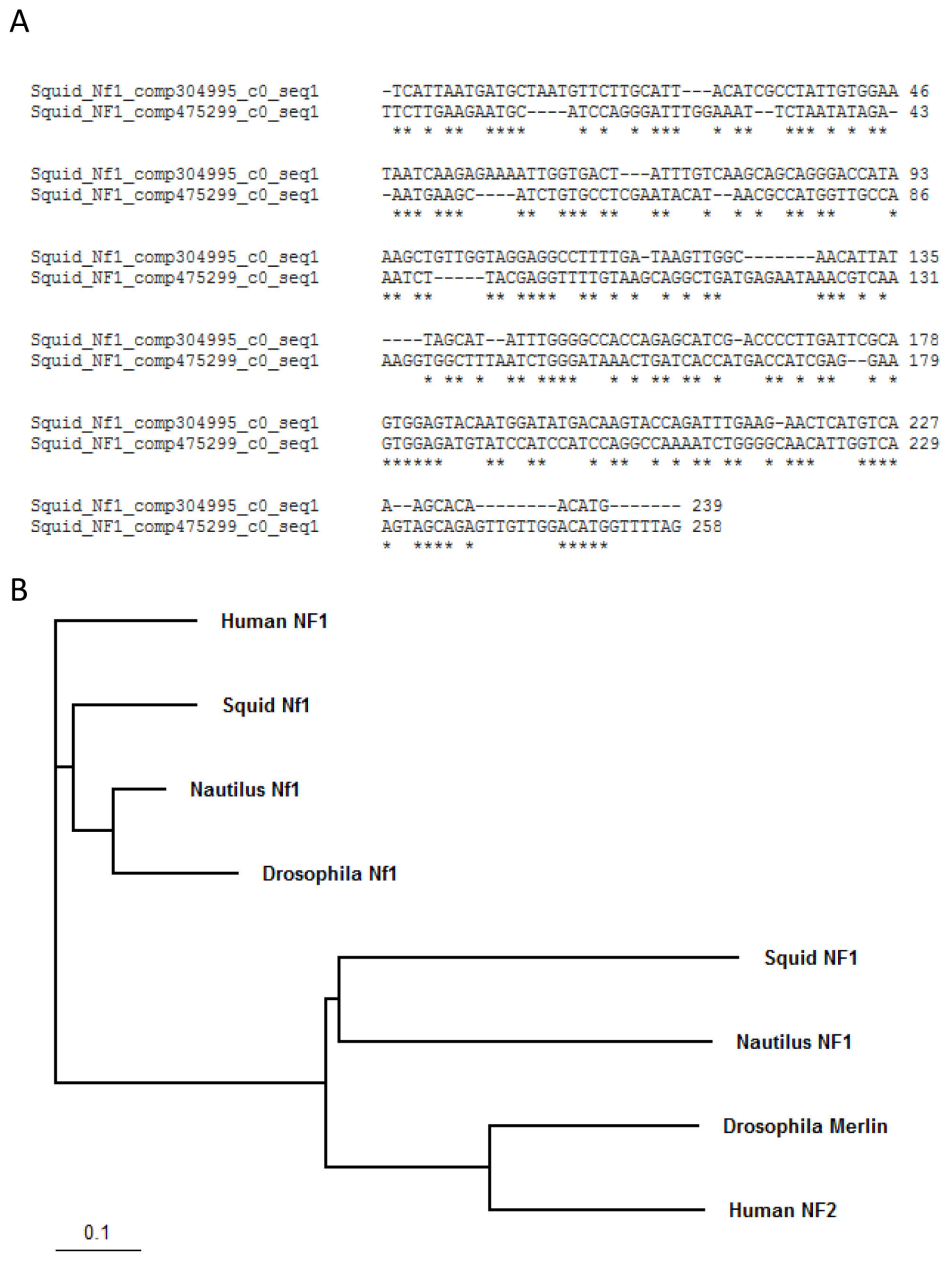
NF1/Nf1 sequence comparisons. A. Nucleotide sequence alignment using ClustalW2 of squid contigs that were found from this analysis indicate potential gene duplication of NF1/Nf1 in cephalopods. Comp304995_c0_seq1 has best hit with Drosophila Nf1 and comp475299_c0_seq1 with Human NF1. Asterisk(*) indicate sequence identity. B. Phylogenetic tree using Human NF1 and NF2, Drosophila Nf1 and Merlin as well as *Nautilus* and squid NF1/Nf1 contigs. Note that Nautilus and squid NF1 contigs, and Nautilus and squid Nf1 contigs cluster together. Bar indicates phylogenetic distance.

Previous studies have shown that there was duplication of part of the genome in the cephalopod lineage [19]. The new genes that were created could be selected to play an advantageous role in eye evolution and potentially to contribute to the formation of the camera-type eye in cephalopods [19]. Based on this result we searched for potential duplication events and subsequent selection of these genes. In order to perform this task we compared the *Nautilus* transcriptome with the combined Human and Drosophila proteome (Nautilus versus Human and Drosophila) and we selected homologous contigs one showing best hit with a Human homologue and the other with the Drosophila homologue. We performed the same analysis with the squid transcriptome (Squid versus Human and Drosophila). Candidate contigs were selected based on the degree of conservation and if the hit was in the same region of the human and fly homologues (Figure 7). The analysis revealed NF1/Nf1 as a potential gene that was duplicated and selected in the cephalopod lineage. There were four contigs expressed in the squid transcriptome from which three had a best hit with the NF1 Human homologue while the other one had a best hit with the Nf1 Drosophila homologue. We found four transcripts to be expressed in the *Nautilus* transcriptome from which two had a best hit with the NF1 Human homologue while the other two had a best hit with the Nf1 Drosophila homologue. We further investigated this hypothesis by comparing the sequences (Table 2). There are two contigs in *Nautilus* and two in squid transcriptome that have a hit in the same region in the Drosophila and Human NF1/Nf1 gene, which provides strong evidence that this gene was duplicated and selected in the cephalopod lineage and is not a product of fragmentation during the de novo assembly (Figure 8A and Text S1). Sequences were also tested against NF2 (Merlin) but no similarity was found (data not shown). This result was also confirmed with phylogenetic tree analysis of the NF1 genes where *Nautilus* and squid NF1 contigs, and *Nautilus* and squid Nf1 contigs clustered together (Figure 8B). NF1/Nf1 gene has been found to be activator of Ras GTPases, to act as tumor suppressor and to be associated with Neurofibromatosis type 1 [20,21]. In the eye, it was shown that mice lacking *Nf1* expression lack lens due to loss of ERK signaling and defective proliferation [22]. The role of Nf1 during eye development in *Drosophila melanogaster* is not known. It is tempting to speculate that the new role(s) of NF1/Nf1 might include the formation of lens in cephalopods. 

**Table 2:** Evidence of NF1 gene duplication.

Transcript	Organism	Best hit	Gene name	Hit length	Same region
comp172849_c0_seq1	Squid	Human	NF1	244-367	
comp180427_c0_seq2	Squid	Human	NF1	1674-1786	
comp304995_c0_seq1	Squid	Drosophila	Nf1	1523-1609	*
comp475299_c0_seq1	Squid	Human	NF1	1572-1656	*
comp6453_c0_seq1	Nautilus	Human	NF1	35-318	
comp83316_c0_seq1	Nautilus	Human	NF1	1597-1919	*
comp34236_c0_seq1	Nautilus	Drosophila	Nf1	613-1053	
comp63708_c0_seq1	Nautilus	Drosophila	Nf1	1501-1987	*

In all, our study is the first to provide such information at the molecular level related to eye evolution in *Nautilus* and pygmy squid and exemplifies the importance of whole transcriptome studies in animal models for eye evolution and evolution in general.

## Supporting Information

Table S1 **GO enrichment analysis of contigs up-regulated at least two-fold in Nautilus and squid samples.**
(XLSX)

Table S2 ***Nautilus* and squid eye-related genes as found by comparisons with Human and Drosophila homologues.**
(XLSX)

Table S3 **Potential candidate gene pairs for lens evolution in squid.**
(XLSX)

Text S1 **Alignment of NF1/Nf1 *Nautilus* contigs that have best hits with the Human and Drosophila NF1/Nf1 protein.**
(TXT)

Figure S1 **Alignment of squid and Nautilus contigs that have best hits with Human and Drosophila CAP1/capt homologues respectively.** Asterisk(*) indicates sequence identity.
(TIF)

Figure S2 **Alignment of squid and Nautilus contigs that have best hits with Drosophila and Human Gef26/RAPGEF2 homologues respectively.** Asterisk(*) indicates sequence identity.
(TIF)

Figure S3 **Alignment of squid and Nautilus contigs that have best hits with Drosophila and Human CG5198/CD2BP2 homologues respectively.** Asterisk(*) indicates sequence identity.
(TIF)
