# Supplementary figures and images for: Transcriptome Analysis of Nautilus and Pygmy Squid Developing Eye Provides Insights in Lens and Eye Evolution
Source: PLoS One. 2013 Oct 16;8(10):e78054. doi: 10.1371/journal.pone.0078054 (PMC3803853; doi:10.1371/journal.pone.0078054)

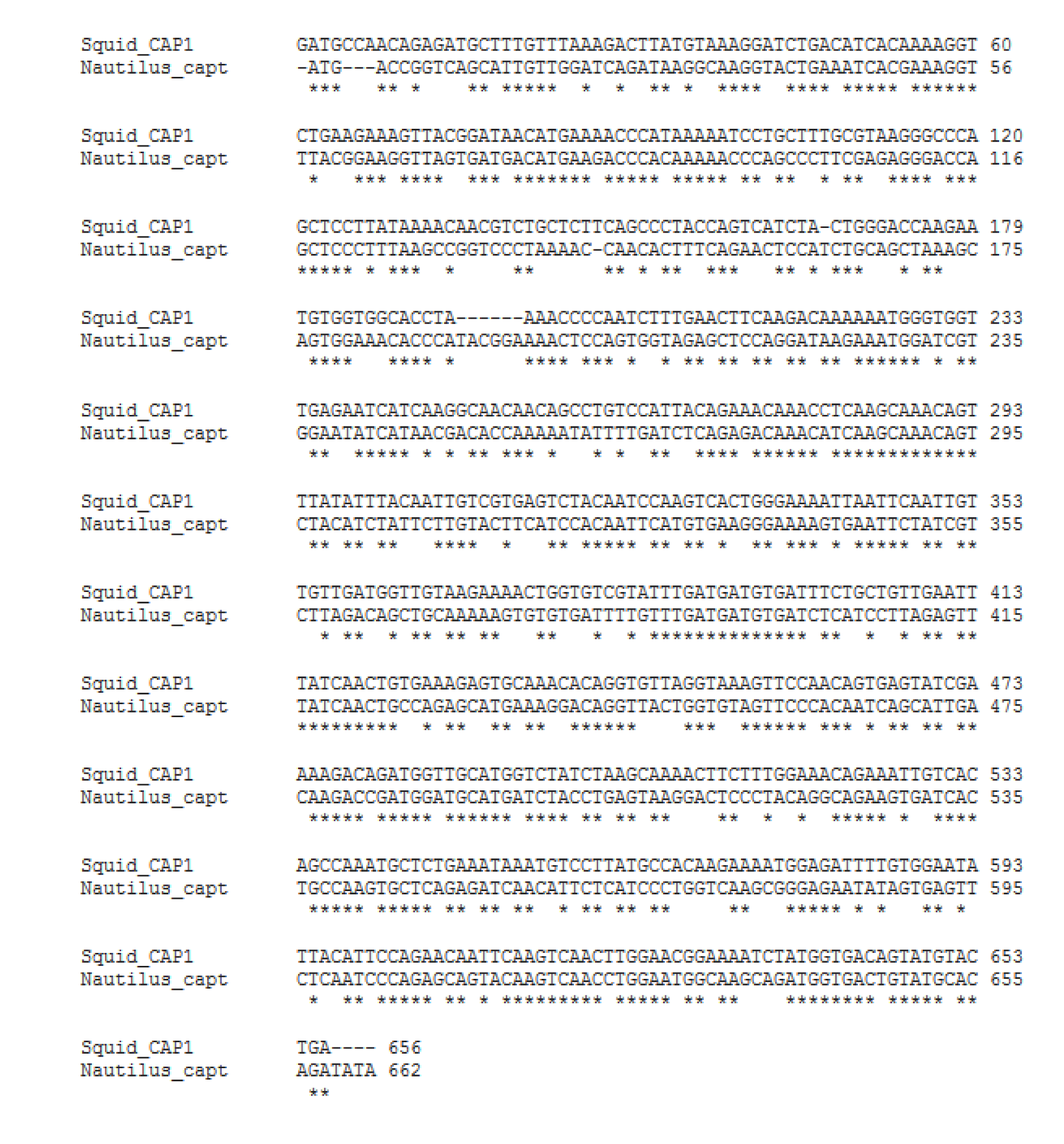

Supplement: Figure S1 — Alignment of squid and Nautilus contigs that have best hits with Human and Drosophila CAP1/capt homologues respectively. Asterisk(*) indicates sequence identity. (TIF) [file pone.0078054.s005.tif]

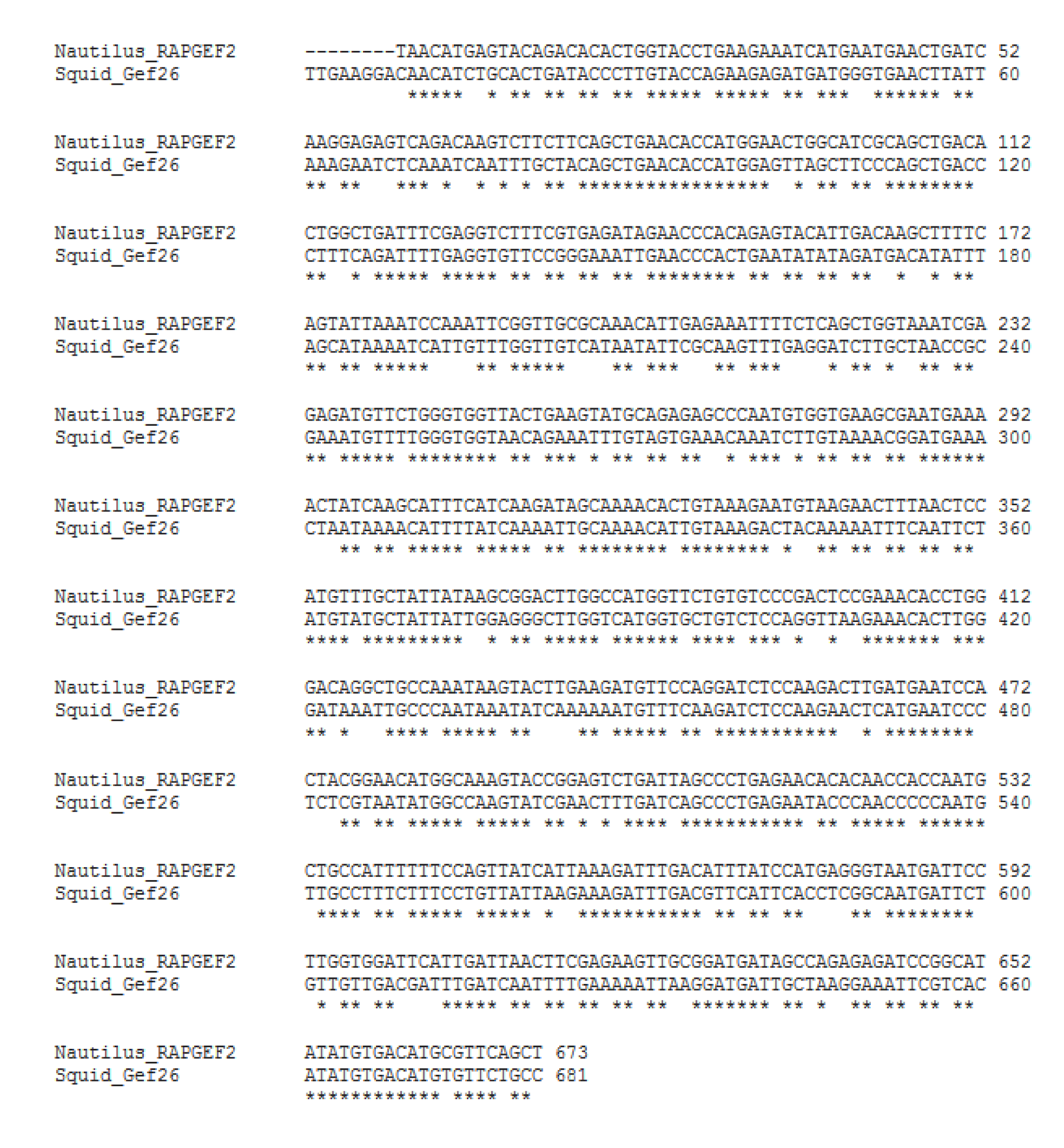

Supplement: Figure S2 — Alignment of squid and Nautilus contigs that have best hits with Drosophila and Human Gef26/RAPGEF2 homologues respectively. Asterisk(*) indicates sequence identity. (TIF) [file pone.0078054.s006.tif]

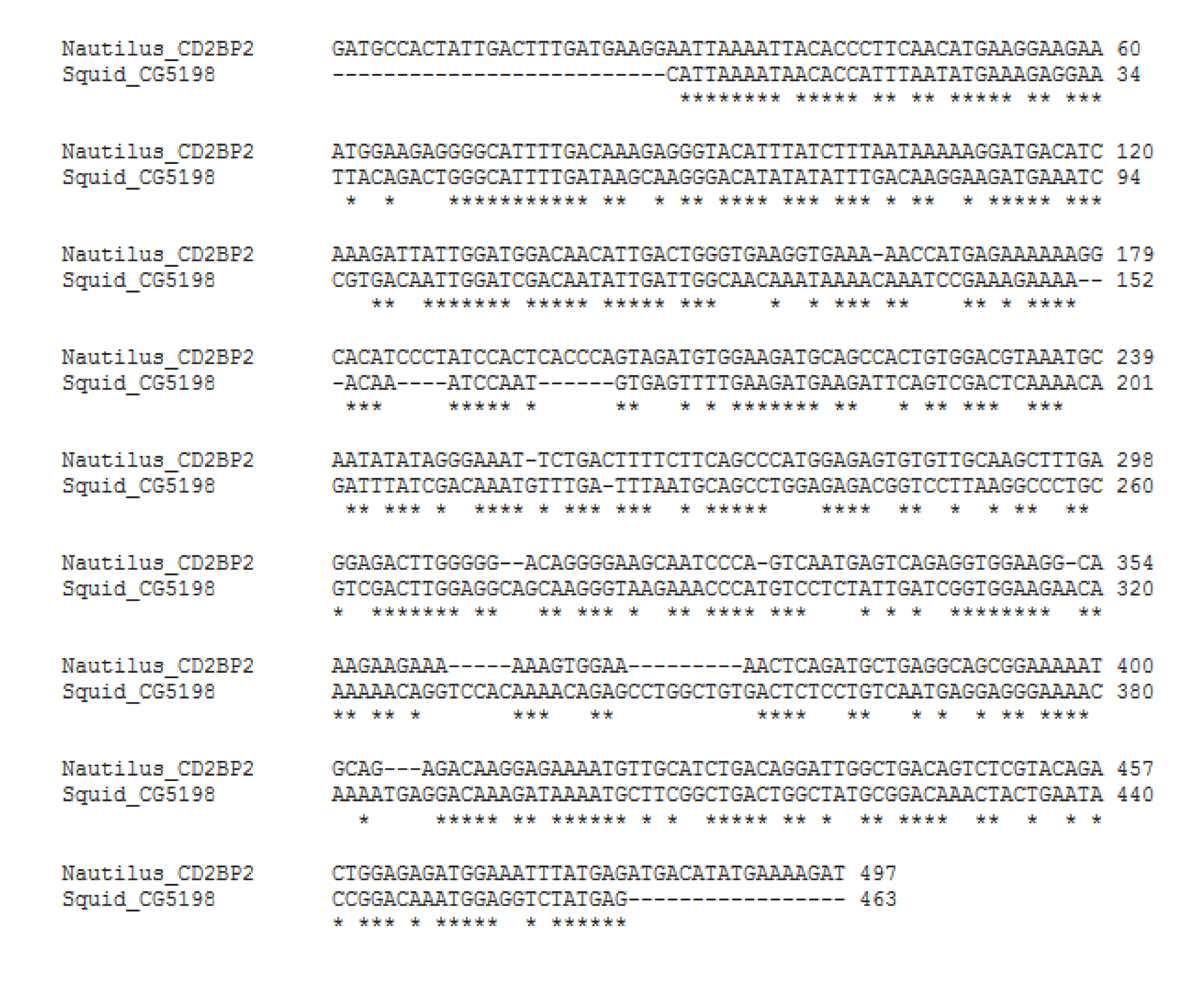

Supplement: Figure S3 — Alignment of squid and Nautilus contigs that have best hits with Drosophila and Human CG5198/CD2BP2 homologues respectively. Asterisk(*) indicates sequence identity. (TIF) [file pone.0078054.s007.tif]
